# Supplementary material for: Obatoclax and Paclitaxel Synergistically Induce Apoptosis and Overcome Paclitaxel Resistance in Urothelial Cancer Cells
Source: Cancers (Basel). 2018 Dec 5;10(12):490. doi: 10.3390/cancers10120490 (PMC6316685; doi:10.3390/cancers10120490)
Supplement: Supplementary file 1 [file cancers-10-00490-s001.pdf]

Supplementary Materials: Obatoclax and Paclitaxel Synergistically Induce Apoptosis and Overcome Paclitaxel Resistance in Urothelial Cancer Cells

Rocío Jiménez-Guerrero, Jessica Gasca, M. Luz Flores, Begoña Pérez-Valderrama, Cristina Tejera-Parrado, Rafael Medina, María Tortolero, Francisco Romero, Miguel A. Japón and Carmen Sáez

Table S1. Patients' clinical characteristics.

Patient	Tumor Grade	TNM	Gender	Age	Mcl-1 Levels	Overall Survival (months)	Relapse (months)
1	G2	TaN0M0	M	73	0	108	
2	G3	T1bN0Mx	M	73	0	24	24 exitus *
3	G1	TaN0M0	M	61	0	100	
4	G3	T1N0M0	M	73	0	120	
5	G1	TaN0M0	M	64	0	94	
6	G1	TaN0M0	F	53	0	96	
7	G2	T1N0M0	M	75	0	24	24 exitus
8	G1	TaN0M0	F	79	0	120	
9	G1	TaN0M0	M	77	0	120	
10	G1	TaN0M0	M	73	0	96	
11	G1	TaN0M0	M	58	0	75	75 exitus *
12	G2	T1N0M0	M	53	0	96	
13	G1	TaN0M0	M	75	0	108	
14	G2	T1N0M0	M	79	0	24	
15	G2	T1aN0M0	M	83	0	60	
16	G1	TaN0M0	M	71	0	96	
17	G1	TaN0M0	M	74	0	96	
18	G1	TaN0M0	M	82	0	85	
19	G1	TaN0M0	M	70	0	110	
20	G1	TaN0M0	M	72	0	100	
21	G1	T1aN0M0	M	71	0	75	75 exitus *
22	G2	TaN0M0	F	74	0	108	
23	G3	T1aN0M0	M	73	0	48	48 exitus
24	G2	TaN0M0	M	70	0	108	
25	G1	TaN0M0	M	43	0	108	96
26	G1	TaN0M0	M	51	0	104	
27	G2	T1aN0M0	M	88	0	24	
28	G1	TaN0M0	M	73	0	104	
29	G1	T1N0M0	M	61	0	106	
30	G3	T1aN0M0	M	69	0	104	
31	G1	TaN0M0	M	80	0	106	
32	G1	TaN0M0	M	50	0	108	
33	G1	TaN0M0	M	57	0	108	
34	G3	T1N0M0	M	55	1	96	
35	G1	TaN0M0	F	85	0	48	
36	G1	TaN0M0	M	57	0	108	
37	G1	TaN0M0	F	61	0	128	

38	G1	TaN0M0	M	69	0	156	
39	G2	TaN0M0	M	72	0	24	24 exitus *
40	G1	TaN0M0	M	66	0	108	
41	G2	TaN0M0	F	72	0	110	
42	G2	TaN0M0	M	72	0	106	94
43	G1	TaN0M0	F	49	0	108	100
44	G2	TaN0M0	M	81	0	48	
45	G2	TaN0M0	M	46	0	108	
46	G2	TaN0M0	F	77	0	96	
47	G2	T1aN0M0	M	75	0	48	48 exitus *
48	G3	T1N0M0	M	64	0	108	
49	G1	TaN0M0	M	65	0	108	
50	G1	TaN0M0	M	62	1	96	
51	G1	TaN0M0	M	74	0	52	52 exitus *
52	G2	T1N0M0	M	74	0	50	50 exitus *
53	G1	TaN0M0	M	51	0	108	
54	G3	T2N1M1	M	71	1	64	64 exitus
55	G3	T3N2M1	M	77	1	20	20 exitus
56	G3	T2N2M1	F	45	0	132	120
57	G3	T2N2M1	F	77	1	36	36 exitus
68	G2	T2NxMx	M	70	0	130	118
59	G3	T2N1M1	M	63	1	20	20 exitus
60	G3	T2N1M1	M	70	1	11	11 exitus
61	G3	T2N1M1	M	41	1	38	38 exitus
62	G3	T2NxM1	M	75	1	15	15 exitus
63	G3	T2N1M1	F	62	1	14	14 exitus
64	G3	T2N0M0	M	81	1	36	
65	G3	T2N0M0	M	46	0	108	
66	G3	T2NxMx	F	84	0	36	36 exitus
67	G3	T2N0Mx	M	69	1	96	
68	G3	T2N0M0	M	73	0	85	
69	G2	T2NxMx	M	71	0	12	12 exitus *
70	G3	T4N1M1	M	80	0	6	6 exitus
71	G3	T2N0M0	M	75	1	15	15 exitus
72	G3	T2N1M1	M	60	1	24	24 exitus

* Exitus non bladder cancer related. Those patients were not included in the Kaplan-Meier analysis.
Mcl-1 levels: Low = 0 and High = 1; TNM: Clinical stage.

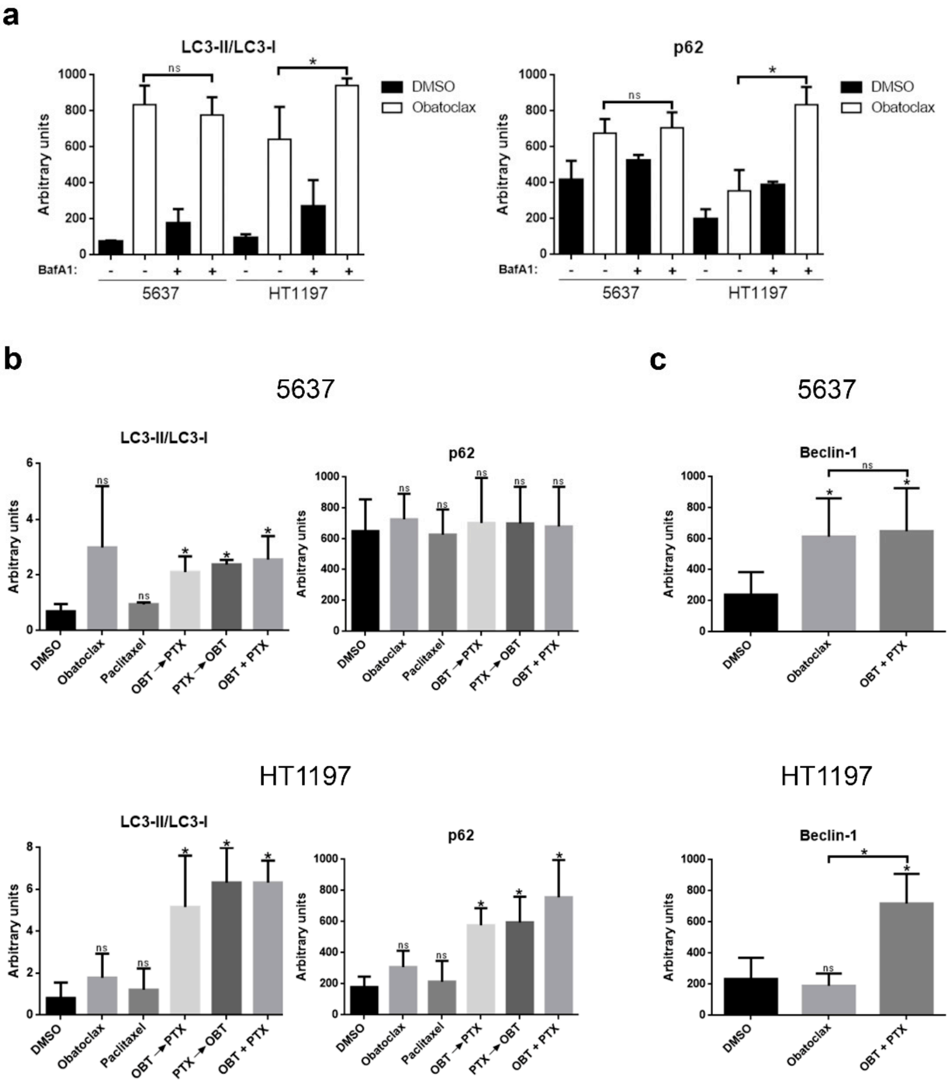

Figure S1. (**a**) Densitometric analysis of LC3-B and p62 proteins in 5637 and HT1197 cells treated with obatoclax in presence or abscense of BafA1. (**b**) Densitometric analysis of LC3-B and p62proteins in 5637 and HT1197 cells treated with paclitaxel and obatoclax combinations. (**c**) Densitometric analysis of beclin-1 protein in 5637 and HT1197 cells treated with obatoclax or obatoclax + paclitaxel.

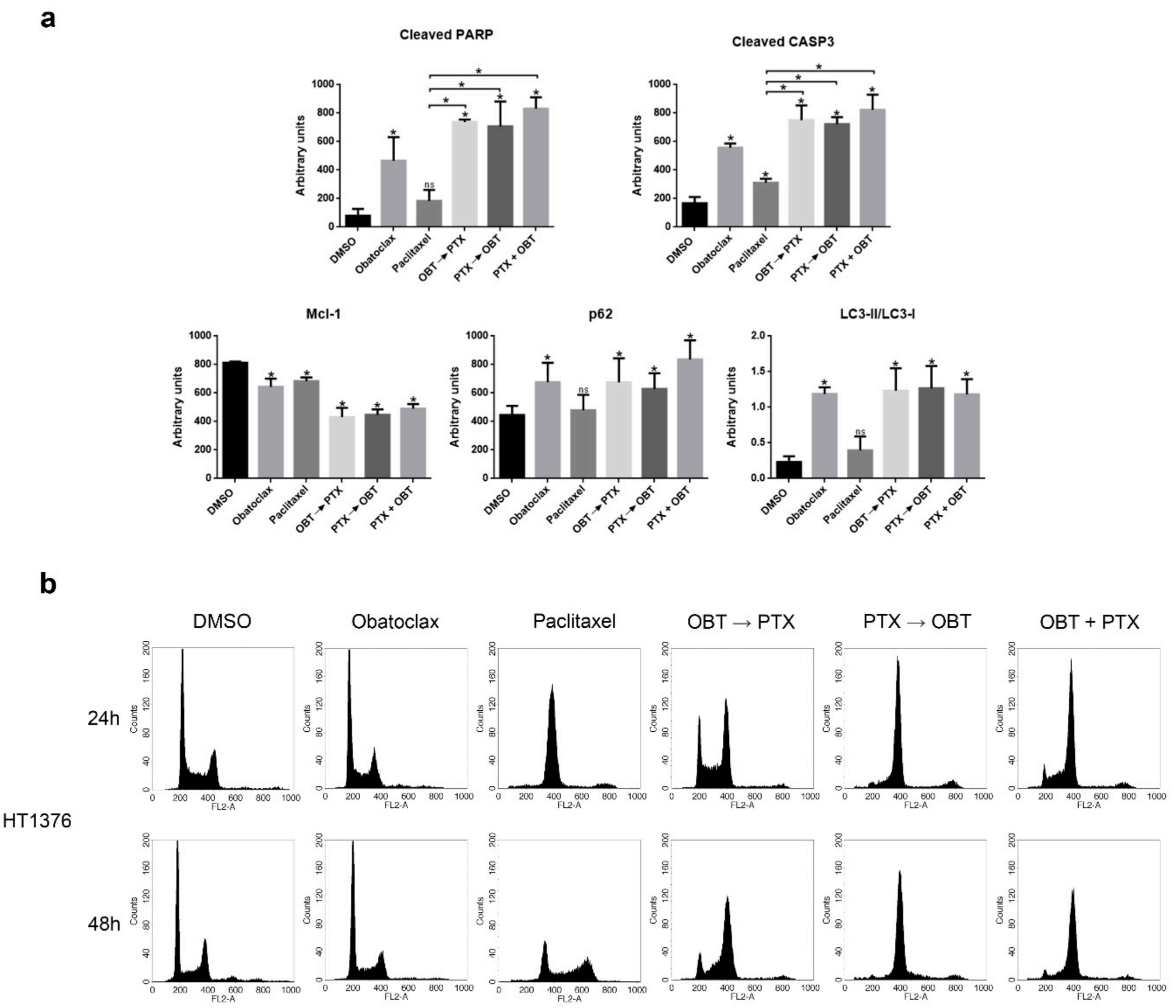

Figure S2. (**a**) Densitometric analysis of cleaved PARP, cleaved CASP3, Mcl-1, p62 and LC3-B in HT1376 cells. (**b**) Cell cycle analysis of propidium iodide-stained cells by flow cytometry.

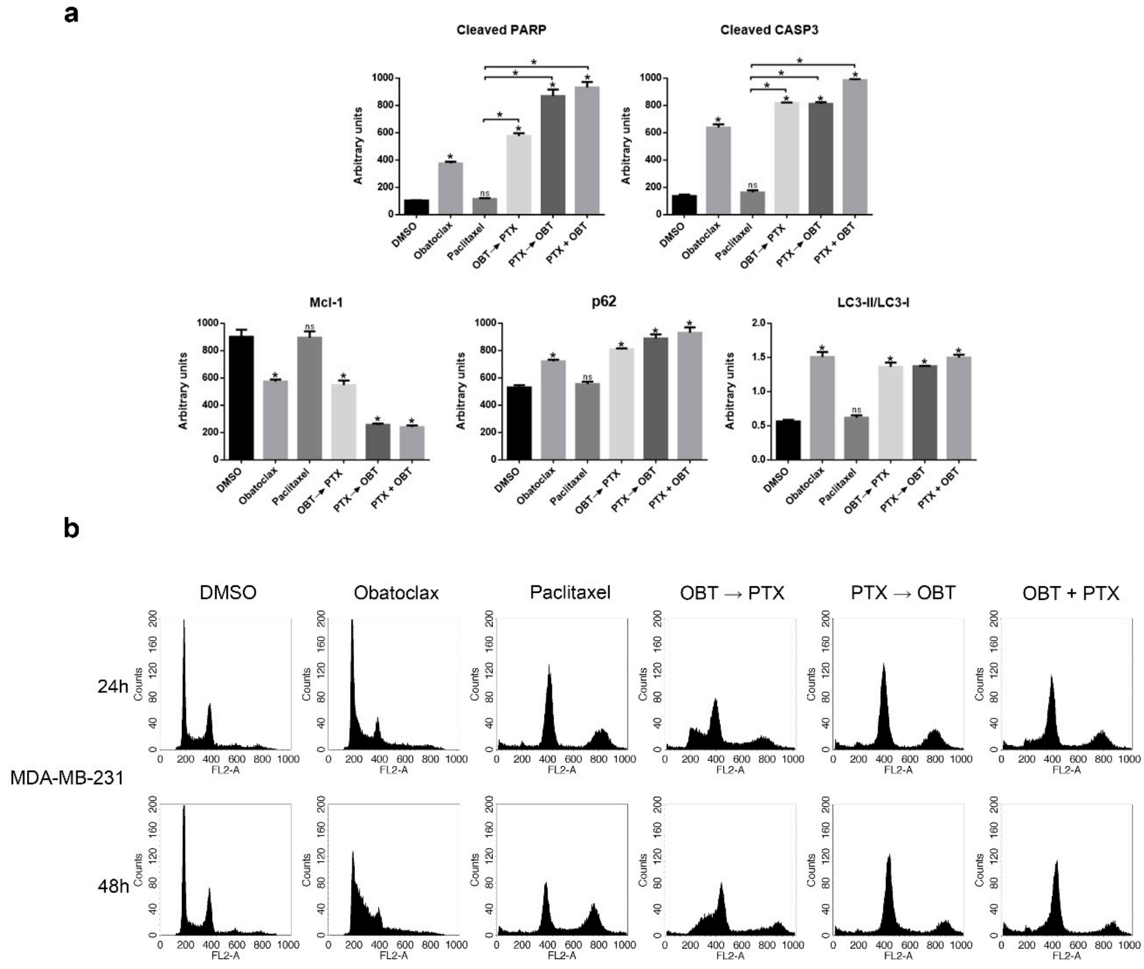

Figure S3. (**a**) Densitometric analysis of cleaved PARP, cleaved CASP3, Mcl-1, p62 and LC3-B in MDA-MB-231 cells. (**b**) Cell cycle analysis of propidium iodide-stained cells by flow cytometry.
